# Glutathione prevents high glucose-induced pancreatic fibrosis by suppressing pancreatic stellate cell activation via the ROS/TGFβ/SMAD pathway

**DOI:** 10.1038/s41419-022-04894-7

**Published:** 2022-05-06

**Authors:** Jitai Zhang, Juan Bai, Qian Zhou, Yuxin Hu, Qian Wang, Lanting Yang, Huamin Chen, Hui An, Chuanzan Zhou, Yongyu Wang, Xiufang Chen, Ming Li

**Affiliations:** 1 Cardiac Regeneration Research Institute, School of Basic Medical Sciences, Wenzhou Medical University, Wenzhou, China; 2 Department of Biochemistry and Molecular Biology, School of Basic Medical Sciences, Wenzhou Medical University, Wenzhou, China; 3 Department of Anesthesia and Critical Care, The Second Affiliated Hospital of Wenzhou Medical University, Wenzhou, China; 4 The Second Affiliated Hospital and Yuying Children’s Hospital of Wenzhou Medical University, Wenzhou, China

**Keywords:** Pre-diabetes, Pre-diabetes

## Abstract

The activation of pancreatic stellate cells (PSCs) is the key mechanism of pancreatic fibrosis, which can lead to β-cell failure. Oxidative stress is an important risk factor for PSC activation. There is no direct evidence proving if administration of glutathione can inhibit fibrosis and β-cell failure. To explore the role of glutathione in pancreatic fibrosis and β-cell failure induced by hyperglycaemia, we established a rat model of pancreatic fibrosis and β-cell failure. The model was founded through long-term oscillating glucose (LOsG) intake and the setup of a sham group and a glutathione intervention group. In vitro, rat PSCs were treated with low glucose, high glucose, or high glucose plus glutathione to explore the mechanism of high glucose-induced PSC activation and the downstream effects of glutathione. Compared with sham rats, LOsG-treated rats had higher reactive oxygen species (ROS) levels in peripheral leukocytes and pancreatic tissue while TGFβ signalling was upregulated. In addition, as the number of PSCs and pancreatic fibrosis increased, β-cell function was significantly impaired. Glutathione evidently inhibited the upregulation of TGFβ signalling and several unfavourable outcomes caused by LOsG. In vitro treatment of high glucose for 72 h resulted in higher ROS accumulation and potentiated TGFβ pathway activation in PSCs. PSCs showed myofibroblast phenotype transformation with upregulation of α-SMA expression and increased cell proliferation and migration. Treatment with either glutathione or TGFβ pathway inhibitors alleviated these changes. Together, our findings suggest that glutathione can inhibit PSC activation-induced pancreatic fibrosis via blocking ROS/TGFβ/SMAD signalling in vivo and in vitro.

## Introduction

According to the statistics of the International Diabetes Federation, the global prevalence of diabetes among the age group of 20–79 years was 9.3% in 2019. Without sufficient interventions to manage this pandemic, the number of people with diabetes worldwide is expected to increase to 578 million people (10.2% of the population) by 2030 and 700 million (10.9%) by 2045 [1]. Without effective and timely interventions, prediabetes, which is the pre-stage of overt diabetes, can result in diabetes. A meta-analysis demonstrated that there is a strong relationship among the consumption of sugar-sweetened beverages and the risk of metabolic syndrome and type 2 diabetes (T2DM) [2]. We previously showed that oral intake of long-term oscillating glucose (LOsG, 4 times/day for 38 days) can lead to β-cells gaining metabolic memory in reactive oxygen species (ROS) stress, inhibition of insulin and SOD-2 expression, and the development of prediabetes with hypoinsulinemia and glucose intolerance [3]. However, the mechanisms underlying LOsG-induced prediabetes remain to be revealed.

T2DM is often accompanied with islet fibrosis in both animal models and patients [4, 5]. Islet fibrosis in T2DM may be an important mechanism of β-cell failure [6, 7]. However, the underlying mechanisms of pancreatic islet fibrosis and β-cell failure remain unclear. Pancreatic inflammation induced by long-term hyperglycaemia and high free fatty acid levels is involved in the pathogenesis of islet fibrosis [8]. In addition, the activation of pancreatic stellate cells (PSCs) is a key mechanism for pancreatic islet fibrosis.

PSCs are myofibroblast-like cells found in the pancreas, accounting for 4–7% of total parenchymal cells in the gland. PSCs express desmin and glial fibrillary acidic protein (GFAP) specifically in pancreas [9]. In normal pancreas, PSCs remain in a metabolically and functionally inactive state, which does not promote fibrosis. Under pathological conditions, such as oxidative stress (OxS), inflammation, and other adverse factors, PSCs can become activated [9, 10]. Once activated, they express α-smooth muscle actin (α-SMA) and synthesize collagen, as well as other extracellular matrix proteins, such as fibronectin [11]. Clinical studies have revealed that PSC activation in T2DM pancreas leads to islet fibrosis [12]. In vitro, PSC activation reduced β-cell insulin secretion and induced β-cell apoptosis, which led to islet functional damage [13]. These findings suggest that pancreatic fibrosis induced by PSC activation may be an important mechanism for β-cell functional damage.

Multiple studies in vivo and in vitro have shown that antioxidants can effectively inhibit pancreatic fibrosis [14–16]. Glutathione (GSH) is a major intracellular antioxidant that plays a key role in reducing the effects of OxS [17]. Clinical studies have reported that the GSH content in erythrocytes of patients with T2DM is reduced [18–20]. As OxS plays an important role in PSC activation and pancreatic fibrosis, we hypothesized that high glucose could stimulate pancreatic cells to produce excess ROS and activate PSCs, that induce pancreatic fibrosis and ultimately lead to β-cell failure. Further, we reasoned that GSH can inhibit PSC activation and proliferation, thereby inhibiting pancreatic fibrosis and protecting islet β cells from damage.

## Methods

### Animals

Thirty female Sprague–Dawley rats weighing 200 ± 10 g were purchased from the Chinese Academy of Medical Sciences (Shanghai, China). The rats were acclimatized in a controlled environment at 22 ± 1 °C with a 12/12 h light/dark cycle, with unlimited access to food and water, for two weeks prior to the experiments. The research protocol was approved by the Institutional Animal Care and Use Committee of Wenzhou Medical University, China. All experiments were conducted according to the guidelines of the committee.

**Fig. 1:**
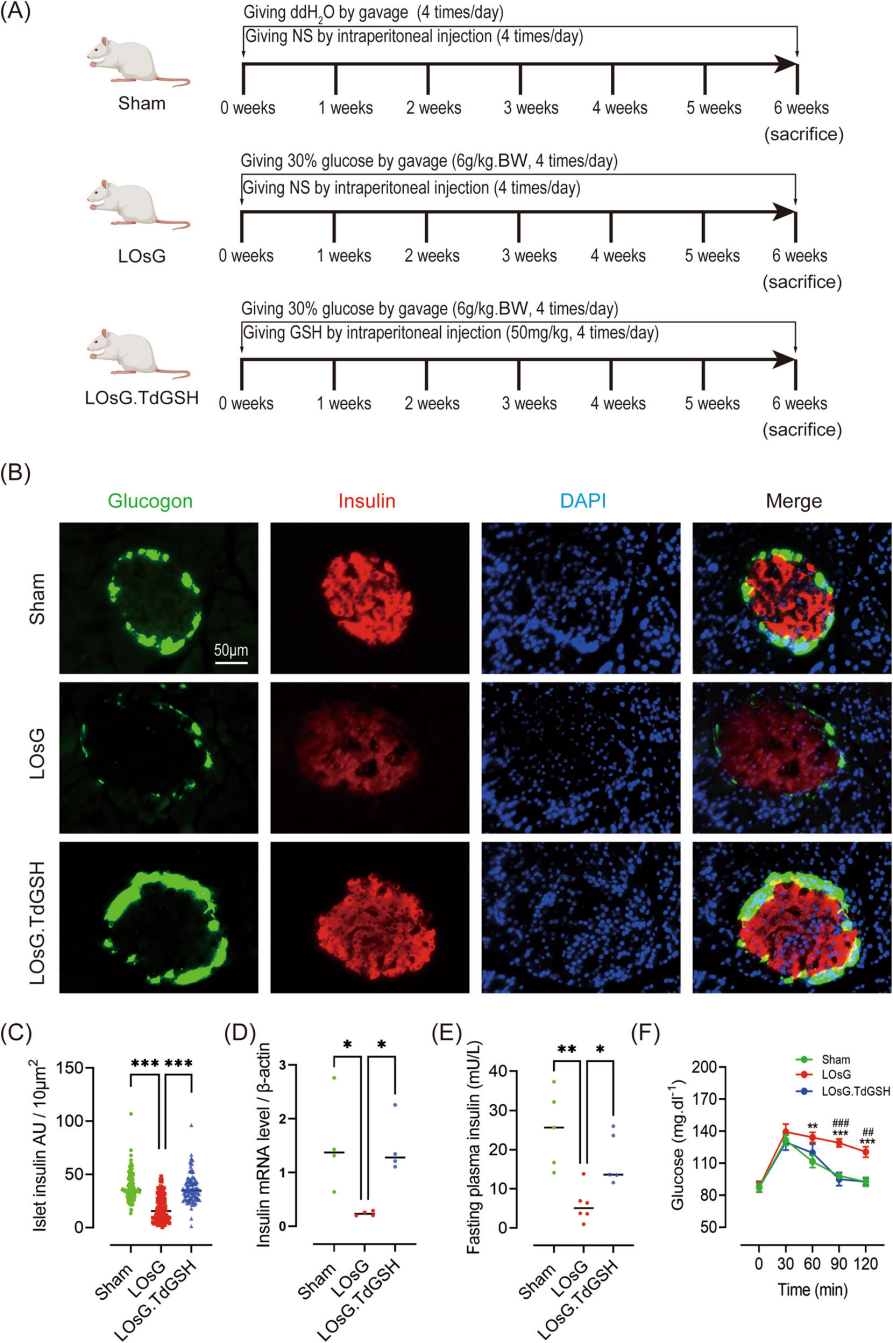
GSH prevents LOsG-induced β-cell failure in vivo. **A** Sprague–Dawley rats were randomly allocated into sham (*n* = 7), LOsG (*n* = 7), and LOsG.TdGSH (*n* = 7) treatment groups. **B** Pancreatic tissue sections were subjected to immunofluorescence staining for insulin (red) and glucagon (green). Nuclei were stained with DAPI (blue). Representative images are shown. **C** Quantification of insulin staining ((*n* = 100 islets/group; presented as arbitrary unit/10 μm^2^). **D** Quantification of insulin mRNA in pancreas (*n* = 4/group). **E** Fasting blood insulin level (*n* = 6/group). **F** Oral glucose tolerance test-based blood glucose curve. Blood samples were collected at 30, 60, 90, and 120 min after glucose oral gavage (*n* = 7/group). Data are shown as mean ± SEM, *P < 0.05, **P < 0.01, ***P < 0.001.

Animals in the LOsG and sham groups (*n* = 10 in both groups) were given 6 g/kg glucose and distilled water (2 mL/100 g body weight), respectively, by gavage every 6 h for 6 weeks. The sample size was calculated by using the *Sample Size Calculator* (https://www.surveysystem.com/sscalc.htm) and met the pre-specified effect size. To evaluate whether LOsG induces ROS homeostasis disorder, a timely intraperitoneal injection of GSH (50 mg/kg/6 h) was made into animals challenged with LOsG as described above (LOsG.TdGSH group, *n* = 10). Treatments were blinded by masking sample labels. Each group was randomized by using the website www.randomization.com. The experimental period was six weeks (Fig. 1a). If animals died unexpectedly in the experiment, the relevant data were excluded.

### Picro-Sirius red staining

Rat pancreatic tissues were fixed in 4% paraformaldehyde (PFA) for 3 h and paraffin-embedded using a standard protocol. Consecutive sections (5 μm) were prepared and mounted on glass slides. The sections were deparaffinized in xylene and rehydrated by washing with graded ethanol (100–70% *v*/*v*) in water. Picro-Sirius Red staining was performed using 0.1% Direct Red in saturated picric acid for 1 h (Sigma-Aldrich, St. Louis, MO, USA). The stained sections were cleared by two dips in 0.5% acetic acid water. Then, the sections were dehydrated with ethanol, mounted, and imaged with Leica fluorescence microscope (Leica DM6000B) using Leica LAS X software. Quantitative analysis was performed using ImageJ software (version 1.60; National Institutes of Health, Bethesda, MD, USA).

### ROS detection

The oxidative fluorescent dye dihydroethidium (DHE; Beyotime, Shanghai, China) was used to detect ROS generation in pancreatic tissue sections. The sections (5 μm) were stained with 5 μM DHE at 25 °C in the dark for 1 h according to the manufacturer’s instructions. ROS generation was detected using a Leica fluorescence microscope (Leica DM6000B) and Leica LAS X software, and the quantitative analysis was performed with ImageJ.

For white blood cells (WBCs), blood samples were collected from rats 1 h before and after glucose gavage (2 g/kg body weight). Red blood cells were removed using a red blood cell lysis buffer (Solarbio, Beijing, China). WBC ROS was detected using a ROS assay kit (Beyotime, Shanghai, China). Briefly, cells were incubated in a serum-free Dulbecco’s modified Eagle’s medium (DMEM) containing 10 µM DCFH-DA at 37 °C for 30 min. After being washed twice with PBS, the WBC ROS were detected using a FACSCanto II flow cytometer (BD, Mountain View, CA, USA).

For cultured PSCs, cells were harvested from 6-well plates after various 96-h treatments. The culture medium was removed, and the cells were incubated with serum-free DMEM containing 10 µM DCFH-DA at 37 °C for 20 min. The cells were washed with serum-free medium three times to remove excess DCFH-DA and immediately analysed using the FACSCanto II flow cytometer. As positive controls, PSCs were pre-incubated with Rosup reagent (Beyotime) for 20 min.

### Immunostaining

Deparaffinized tissues were unmasked using antigen unmasking solution (Vector Laboratories, Burlingame, CA, USA) in a microwave oven. After blocking in 5% goat serum (Beyotime) for 30 min, the sections were incubated with antibodies against insulin, glucagon, fibronectin, desmin, and α-SMA (Supplementary Table 2) at 4 °C overnight. Following a washing step, Alexa Fluor 594-conjugated goat anti-rabbit IgG (1:400, Molecular Probes, Waltham, MA, USA) and/or Alexa Fluor 488-conjugated goat anti-mouse IgG (1:400, Molecular Probes) were/was added as the secondary antibody and DAPI was used for nuclear staining.

For the immunostaining of cultured PSCs, cells were fixed in 4% PFA for 15 min followed by a 15-min incubation in 0.5% Triton-X100. Then, the cells were subjected to immunofluorescence staining for α-SMA, desmin, GFAP, and vimentin (Supplementary Table 2) by an overnight incubation with primary antibodies at 4 °C, followed by a 1-h incubation with secondary antibody (1:500, Molecular Probes) after a wash step. Negative control sections were incubated with PBS instead of primary antibody. Antibodies were detected under a Leica DM6000B fluorescence microscope using Leica LAS X software.

### Western blotting

Total protein was extracted from paraformaldehyde-fixed, paraffin-embedded pancreatic tissues using the FFPE Total Protein Extraction Kit (Sangon Biotech, Shanghai, China) or from cultured PSCs using RIPA Lysis Buffer (Beyotime). Protein concentrations were determined using an enhanced BCA protein assay kit (Beyotime). The proteins (20 μg) were resolved by SDS-PAGE and blotted onto polyvinylidene fluoride membranes. The membranes were blocked in QuickBlock™ Blocking Buffer for 15 min (Beyotime) and then probed with primary antibodies against fibronectin, collagen I, GFAP, α-SMA, TGFβ1, SMAD3, phospho-Smad3 (Thr8), SMAD4, α-tubulin, and β-actin (Supplementary Table 2). HRP-conjugated goat anti-mouse IgG or anti-rabbit IgG (Boster, Wuhan, China) was used as the secondary antibody. Protein band intensities were quantified using ImageJ software (version 1.60).

### Isolation and culture of rat PSCs

PSCs were isolated from female Sprague–Dawley rats, as reported previously [21]. Briefly, pancreatic tissue was digested in Gey’s balanced salt solution containing 0.75 mg/mL collagenase P (Sigma-Aldrich), 0.03% protease (Roche, Mannheim, Germany), and 0.01% DNase I (Roche). The digest was filtered through a 70-μm mesh and centrifuged using a 28.7% Nycodenz gradient (Nycomed PharmaAS, Oslo, Norway). PSCs were collected at the interface between the Nycodenz and the medium. The cells were plated in DMEM containing 10% foetal bovine serum (FBS) and 5.5 mM glucose. This method yielded PSCs with >90% purity and negligible contamination by other cells. Unless stated otherwise, PSCs were preincubated in DMEM containing 10% FBS and 5.5 mM glucose for 24 h. Then, the cells were treated with low (5.5 mM) or high (30 mM) glucose concentrations with or without GSH (4 mg/mL) for 72 h. In some experiments, the cells were additionally treated with 5 ng/mL TGFβ1 (PeproTech Cranbury, NJ, USA) or 10 μM SB431542 (Selleck chemicals, TX, USA).

### Human pancreatic stellate cell (hPSC) culture and treatments

HPSCs (Fenghui Biotechnology Co., Ltd., Changsha, China) were cultured in RPMI 1640 medium (Gibco) with glucose (5.5 mmol/L glucose) supplemented with 10% FBS (Gibco) in an incubator with 5% CO2 at 37 °C. The cells were cultured in normal-glucose medium for 48 h and then treated with the following media: (1) low glucose group (LG) (5.5 mM); (2) high glucose group (HG) (30 mM); (3) HG + GSH (4 mg/mL); (4) LG + TGFβ1 (10 ng/mL); and (5) HG + SB431542 (10 μM). Cells in each group were cultured for 72 h for further study.

### Oil red O staining

PSCs were fixed in 4% PFA at room temperature for 15 min. After three washes with PBS, the cells were incubated with an Oil red O solution (Sigma-Aldrich) for 10 min. Then, cells were washed with PBS three times. Finally, the cells were covered with antifade mounting medium containing DAPI. Images were acquired using the Leica DM6000B fluorescence microscope.

### Quantitative real-time PCR (qRT-PCR)

Total RNA was isolated from pancreatic tissues and cultured PSCs using TRIzol reagent (Beyotime). One milligram of total RNA was reverse-transcribed into cDNA using a Transcriptor First Strand cDNA Synthesis Kit (Roche, Indianapolis, IN, USA). qPCRs were run in an ABI StepOne Plus Real-Time PCR system (Applied Biosystems, Foster City, CA, USA), using SYBR-Green Supermix (Roche). Expression levels of insulin, TGF-β1, α-SMA, and collagen I were normalized to that of β-actin and expressed relative to that in the sham group [22]. The primers used are listed in Supplementary Table 1.

### Apoptosis assay

Apoptosis was detected using the Annexin V-FITC Apoptosis Detection Kit (Beyotime) according to the manufacturer’s instructions. Briefly, PSCs were harvested on day 4. After two washes with cold PBS, the cells were suspended in 195 µL of binding buffer. After the addition of 5 µL of Annexin V-FITC, the cells were incubated at room temperature for 15 min. Then, 10 µL of propidium iodide were added and the cells were further incubated for 10 min. The cells were analysed immediately using the FACSCanto II flow cytometer.

### 5-Ethynyl-2′-deoxyuridine (EdU) incorporation assay

DNA synthesis was assayed using the BeyoClick™ EdU Cell Proliferation Kit (Beyotime) according to the manufacturer’s instructions. PSCs were isolated and cultured in 6-well plates for 48 h. After incubation with 10 μM ethynyl-2′-deoxyuridine solution at 37 °C for 2 h, the cells were harvested and fixed with 4% PFA for 15 min. Then, Azide 594 working solution was added for fluorescent colour reaction. The FACSCanto II flow cytometer was used for fluorescence detection.

### Cell proliferation assay

PSCs were seeded in a 96-well plate at confluency levels of 2000/well and in 100 μL of DMEM. Following 24, 48, 72, or 96 h after seeding, the medium was supplemented with 10 μL of CCK-8 reagent (Beyotime) and the cells were incubated at 37 °C for 2 h. Then, the optional density at 450 nm was measured using a microplate reader (Liuyi, Beijing, China).

### Wound healing assay

Isolated PSCs were cultured in 6-well plates for 72 h. The cell monolayer was scratched with a 200-μL pipette tip. At 0 h and 24 h, images were acquired in five fields of view to determine cell migration. The cell migration rate was calculated as the ratio of diminishing scratch area to total scratch area (normalized to 0 h).

### Statistical analysis

Treatment and data analyses were blinded by masking sample labels. Statistical analyses were performed using GraphPad Prism 7. All data are presented as the mean ± standard error of the mean (SEM). The homogeneity of variances was tested using the F test. One-way ANOVA was used to compare means of three or more groups. Pearson correlation was used to analyse correlations. A value of *P* < 0.05 was considered statistically significant.

## Results

### GSH prevents LOsG-induced β-cell failure in vivo

To simulate the high glucose food intake habits of humans, we administered an extra intake of 6 g/kg d-glucose in female rats every 6 h per day for 6 weeks (Fig. 1a). We first validated the LOsG model by assessing fasting blood glucose levels and oral glucose tolerance testing. As shown in Fig. 1f, 6 weeks of glucose administration did not alter fasting blood glucose levels, but it did impair glucose tolerance (*P* < 0.001). Next, we evaluated insulin expression by immunofluorescence and qRT-PCR. The data showed that LOsG induced a significant reduction in insulin expression at the protein and mRNA levels (*P* < 0.001, *P* < 0.05 respectively, Fig. 1b–d). Additionally, LOsG decreased the fasting plasma insulin level (*P* < 0.01, Fig. 1e). These results indicated that LOsG impaired β-cell function and glucose tolerance. However, timely administration of GSH completely blocked the glucose-induced β-cell damage, as indicated by the rescue of LOsG-induced suppression of insulin expression (*P* < 0.05, Fig. 1b–d), the fasting plasma insulin levels (*P* < 0.05, Fig. 1e), and the glucose tolerance to a normal level (*P* < 0.01, Fig. 1f).

### GSH inhibits pancreatic fibrosis induced by LOsG in vivo

**Fig. 2:**
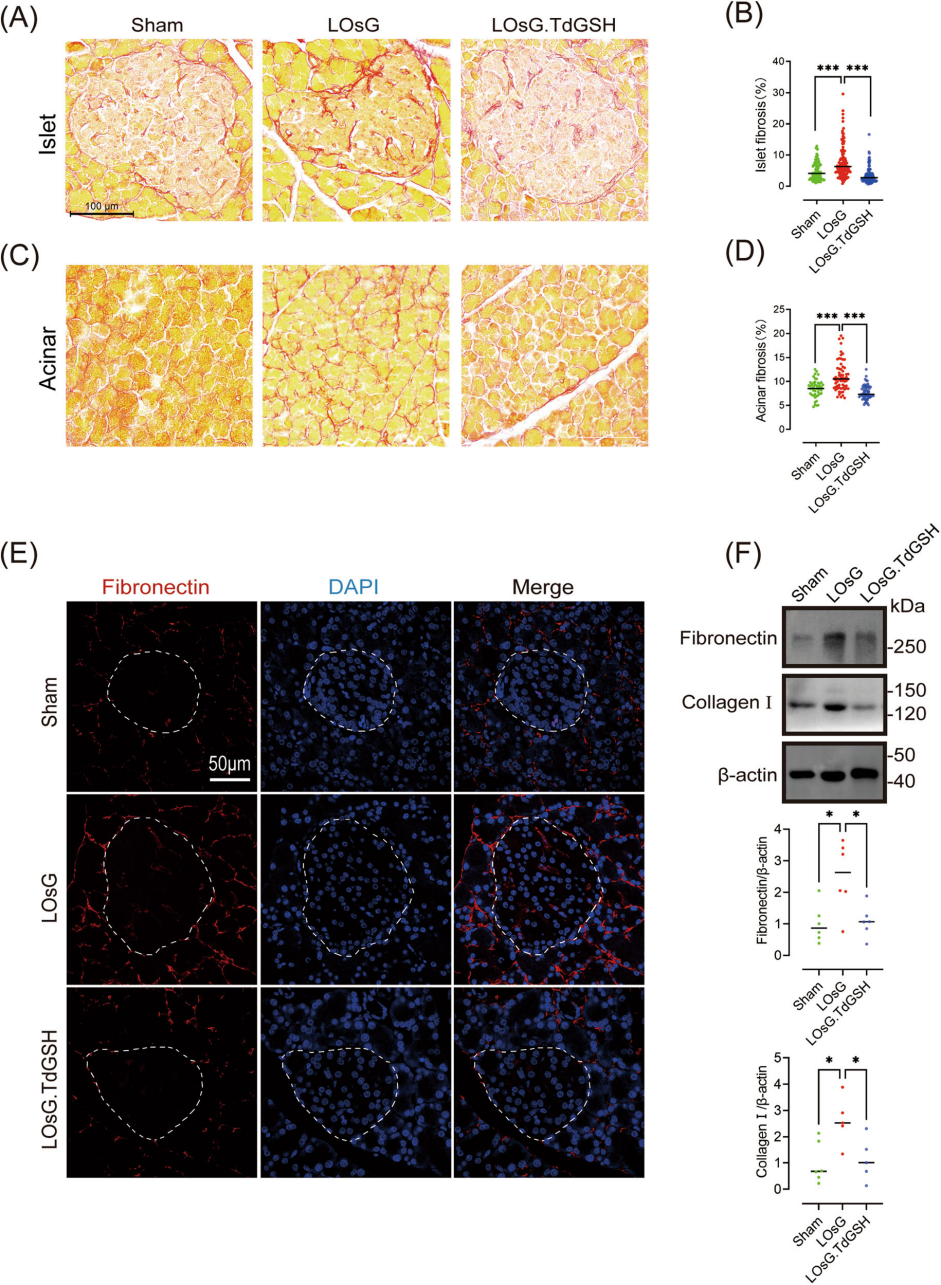
GSH prevents LOsG-induced pancreatic fibrosis in vivo. Representative images of Picro-Sirius red staining and quantification of fibrosis in islet (**A**, **B**) and acinar (**C**, **D**) tissues, respectively. **E** Representative images of fibronectin immunofluorescence staining (red). Nuclei were stained with DAPI (blue). The islets are circled by a dotted line. **F** Western blot data of pancreatic fibronectin and collagen I levels (relative to their levels in sham rats, *n* = 6/group). Data are shown as mean ± SEM, *P < 0.05, ***P < 0.001.

Considering that pancreatic fibrosis may accelerate β-cell destruction [23], we detected fibrosis-related indicators by Picro-Sirius red staining, western blotting, and immunofluorescence. As demonstrated by Picro-Sirius red staining, pancreatic fibrosis was significantly elevated in LOsG-treated rats (*P* < 0.001). However, GSH treatment prevented the increase in fibrosis (*P* < 0.001, Fig. 2a–d). Consistent herewith, the production of collagen I and fibronectin, which are extracellular matrix (ECM) proteins, was clearly increased in LOsG-treated rats (*P* < 0.05), and this trend was suppressed by GSH (*P* < 0.05, Fig. 2e, f).

### GSH prevents LOsG-induced pancreatic cell ROS production in vivo

**Fig. 3:**
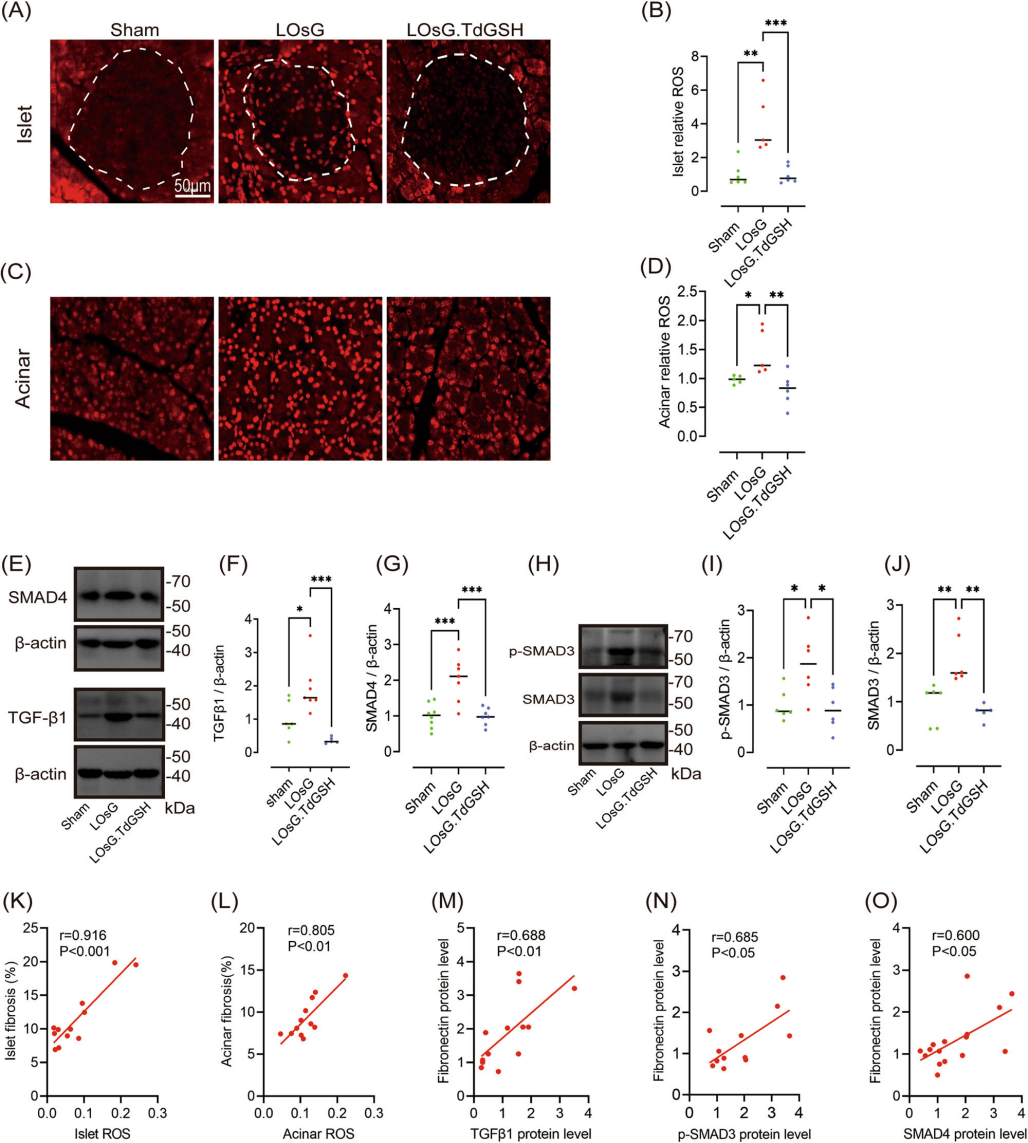
GSH blocks the upregulation of ROS/TGFβ/SMAD signalling induced by LOsG in vivo. Representative images of ROS staining and quantification of ROS staining in islet (**A**, **B**) and acinar (**C**, **D**) tissues. The islets are circled by a dotted line (levels are relative to those in sham rats, *n* = 5–6/group). **E**–**J** Western blot data of pancreatic TGFβ1, SMAD4, p-SMAD3, and SMAD3 (levels are relative to those in sham rats, *n* = 6/group). Correlations between fibrosis and islet ROS (**K**) or acinar ROS (**L**, *n* = 12). Correlations between the fibronectin protein level and TGF-β1 (**M**) or p-SMAD3 (**N**) or SMAD4 (**O**) protein levels were analysed using Pearson’s correlation test (*n* = 12). *P < 0.05, **P < 0.01, ***P < 0.001.

Compared with healthy controls, T2DM patients show ROS accumulation in blood monocytes [24]. To verify whether LOsG can trigger this condition, we collected WBCs from rats 1 h before and after oral 2 g/kg glucose challenge and evaluated ROS accumulation by flow cytometry. ROS accumulation after the 1-h glucose challenge was significantly higher in the LOsG group than that in the sham group (*P* < 0.001, Fig. S1). Interestingly, ROS production in rats of the LOsG.TdGSH group was resembling that in sham rats (*P* > 0.05, Fig. S1), suggesting that GSH prevented LOsG-induced excess ROS production in WBCs. Next, we investigated ROS accumulation in pancreatic cells. Compared with sham rats, LOsG-treated rats showed a 4-fold increase in ROS production in islet cells (*P* < 0.01, Fig. 3a, b) and a 1.45-fold increase in acinar cells (*P* < 0.05, Fig. 3c, d), indicating that β-cells have a poor capability to handle ROS accumulation. To explore whether GSH could eliminate the excess ROS production in response to high glucose, we administered GSH in a timely manner, followed by LOsG treatment. There was no difference between LOsG.TdGSH and sham rats in terms of ROS production (*P* > 0.05, Fig. 3a–d), indicating that GSH prevented LOsG-induced ROS production in pancreatic cells. In addition, pancreatic fibrosis was positively correlated with ROS production (Fig. 3f, g).

### GSH prevents LOsG-induced pancreatic fibrosis via the TGFβ/SMAD pathway in vivo

Considering the crucial role of TGF-β1 in organ fibrosis, we examined whether LOsG contributed to TGF-β1 expression by detecting TGF-β1 protein levels in pancreatic tissues using western blotting. TGF-β1 protein levels were significantly increased in LOsG rats when compared with sham rats (*P* < 0.05, Fig. 3e, f). In addition, TGF-β1 downstream molecules, including SMAD4, SMAD3, and p-SMAD3, were significantly increased in pancreatic tissues of rats in the LOsG group (*P* < 0.001, *P* < 0.05, *P* < 0.01 respectively, Fig. 3e, g–j). Both p-SMAD3 and SMAD3 were upregulated in the LOsG group (Fig. 3h–j), but there was no significant difference in the ratio of p-SMAD3 to SMAD3 (data not shown). We next evaluated the effect of GSH on TGF-β1 signalling activation. GSH effectively prevented TGFβ/SMAD signalling activation by LOsG (Fig. 3e–j). The protein levels of TGF-β1, p-SMAD3, and SMAD4 were positively correlated with fibronectin (*P* < 0.01, *P* < 0.05, *P* < 0.05 respectively, Fig. 3k–o), which is a fibrotic ECM factor. Together, these findings indicated the role of TGFβ/SMAD signalling in the induction of pancreatic fibrosis.

### GSH inhibits PSC activation and proliferation induced by LOsG in vivo and in vitro

**Fig. 4:**
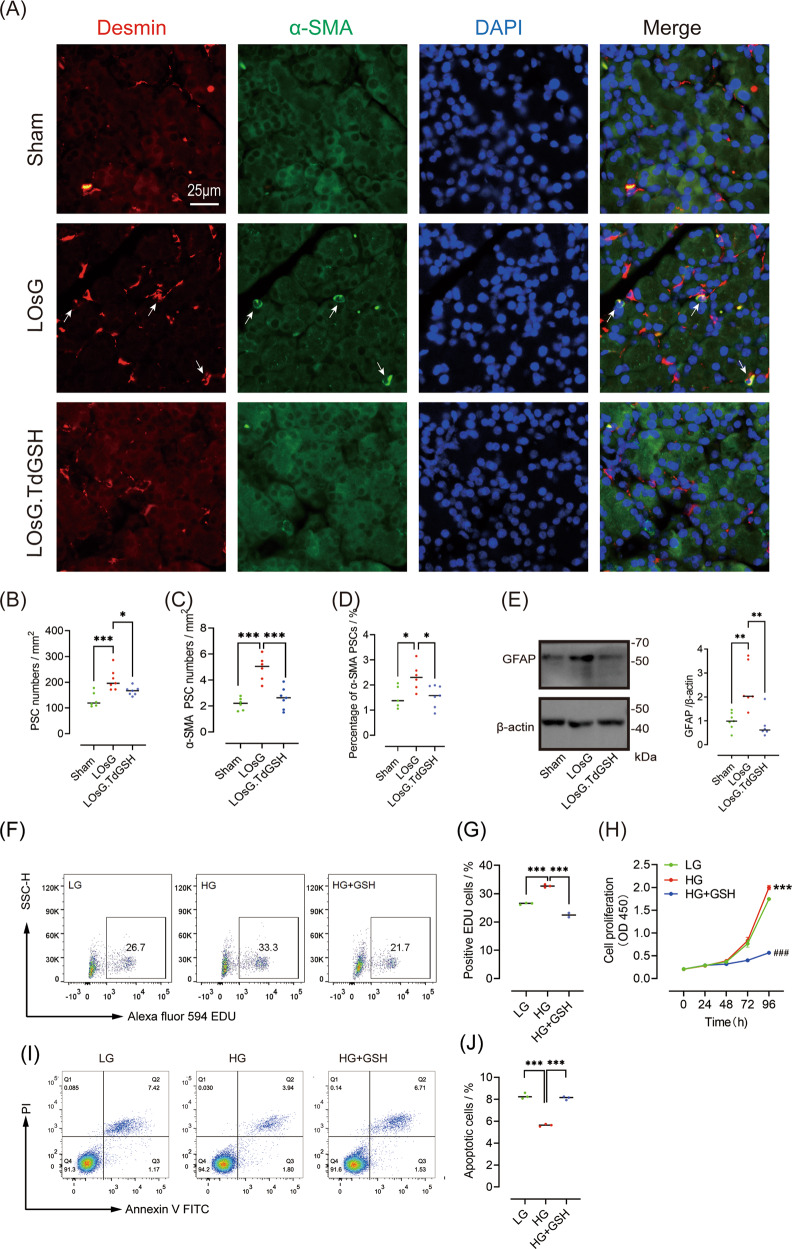
GSH inhibits PSC activation and proliferation induced by LOsG in vivo and in vitro. **A** Representative images of desmin (red) and α-SMA (green) staining. Nuclei were stained with DAPI (blue). The white arrow indicates cells positive for both α-SMA and desmin. Quantification of total PSCs (**B**), α-SMA-positive PSCs (**C**), and percentage of α-SMA-positive PSCs (**D**, *n* = 6/group; presented as pcs/mm^2^). **E** Western blot data of pancreatic GFAP (levels are relative to those in sham rats, *n* = 6/group). **F**–**H** Representative and statistical data of cell proliferation detected by EDU staining and the CCK-8 assay. **I**, **J** Representative and statistical data of cell apoptosis detected by flow cytometry. *P < 0.05, **P < 0.01, ***P < 0.001.

PSC activation is a critical event in pancreatic fibrosis, and activated PSCs have characteristics such as high proliferation and increased ECM secretion. To explore whether LOsG induced PSC activation and proliferation, we detected the expression of desmin and α-SMA using immunofluorescence. We found that LOsG increased not only the total number of PSCs (*P* < 0.001, Fig. 4a, b), but also the number of α-SMA-positive PSCs (*P* < 0.01, Fig. 4c). Further analysis revealed that the proportion of α-SMA-positive PSCs to total PSCs was significantly increased in rats of the LOsG group (*P* < 0.05, Fig. 4d). In addition, the expression of GFAP, another PSC biomarker, was elevated upon LOsG treatment (*P* < 0.01, Fig. 4e). As expected, GSH prevented the increments in total and activated PSCs induced by LOsG (*P* < 0.05). Consistent with the findings in vivo, high glucose increased the proliferation and suppressed apoptosis of PSCs in vitro, whereas GSH prevented these effects (Fig. 4f–j).

### GSH inhibits PSC activation induced by high glucose via the ROS/TGFβ/SMAD pathway in vitro

**Fig. 5:**
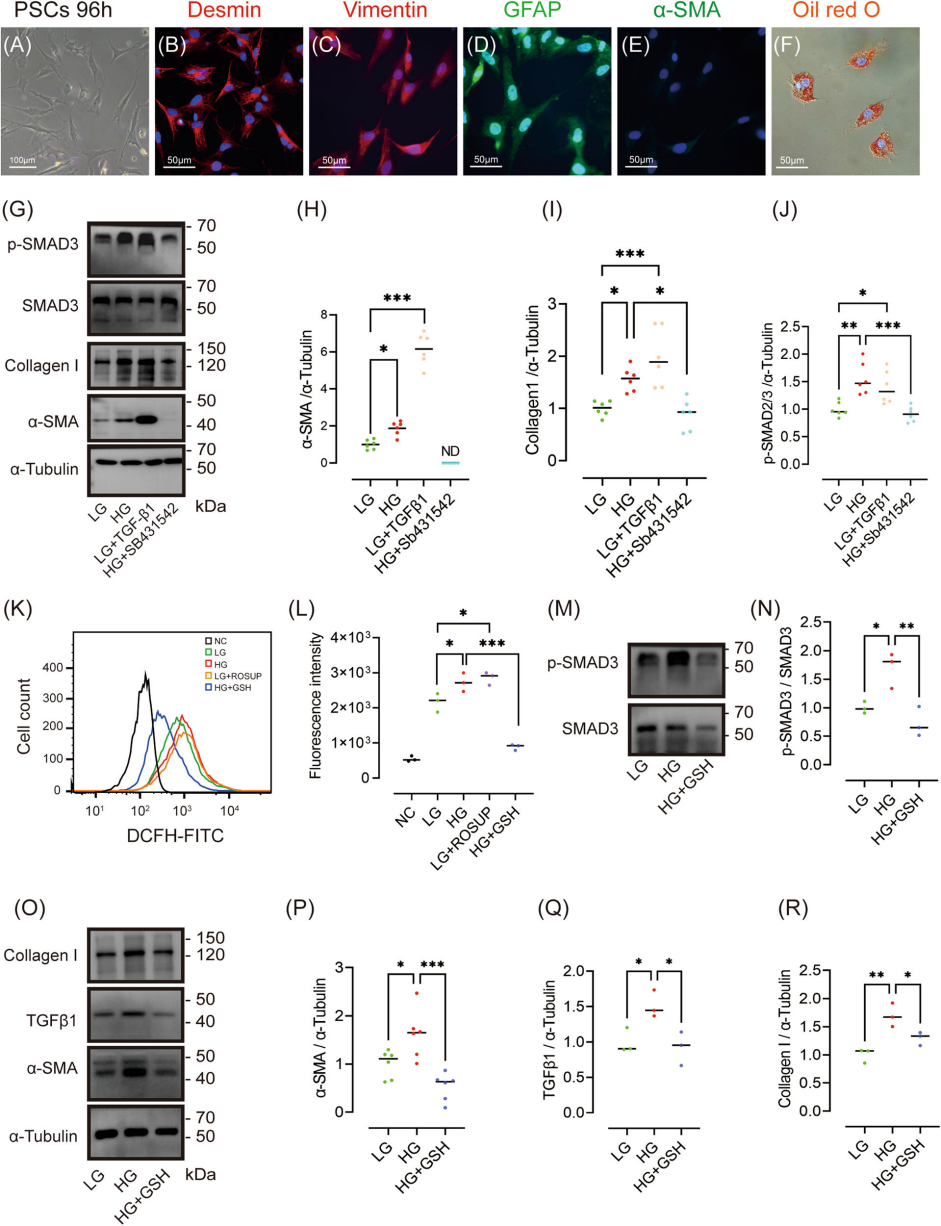
GSH inhibits high glucose-induced PSC activation via the ROS/TGFβ/SMAD pathway in vitro. **A**–**F** Identification and morphological characterization of isolated PSCs cultured in low-glucose medium at 96 h. **A** Morphology of isolated cultured PSCs subjected to immunofluorescence staining for desmin (**B**), vimentin (**C**), GFAP (**D**), and α-SMA (**E**). Lipid droplets were stained with Oil red O (**F**), and nuclei with DAPI (blue). **G**–**J**, **M**–**R** Western blot data of α-SMA, collagen I, SMAD3, p-SMAD3, and TGFβ1 (levels are relative to those in LG group, *n* = 3 or 6, each group). **K**, **L** ROS detected in cultured PSCs (*n* = 3/group). *P < 0.05, **P < 0.01, ***P < 0.001.

The above results indicated an increase in pancreatic fibrosis in rats treated with LOsG, which was accompanied by an increase in ROS/TGFβ/SMAD signalling, and PSC proliferation and activation, which were all prevented by GSH. We speculated that GSH may inhibit high glucose-induced PSC activation via the ROS/TGFβ/SMAD pathway, thereby inhibiting fibrosis progression. Therefore, we evaluated changes in the expression of ROS/TGFβ/SMAD pathway molecules and the effect of GSH thereon in cultured primary PSCs. Figure 5a shows the morphology of PSCs 96 h after isolation. After 96 h of culture in a low-glucose medium, isolated PSCs expressed desmin, vimentin, and GFAP (Fig. 5b–d), but were negative for α‐SMA (Fig. 5e). The cells had dense lipid droplets surrounding the nucleus (Fig. 5f). These findings indicated that the PSCs were not activated in low-glucose medium during the experimental period. Western blot analysis revealed increased expression of α-SMA and collagen I, a marker of PSC activation, in the high-glucose group (*P* < 0.05, Fig. 5g–i). To evaluate the role of TGFβ signalling in high glucose-induced PSC activation, we treated cells in the low-glucose group with 5 ng/mL TGFβ1 and cells in the high-glucose group with 10 μM of the TGFβ pathway inhibitor SB431542. After activation of the TGFβ pathway in PSCs cultured in low glucose, the expression levels of α-SMA and collagen I were significantly upregulated. After TGFβ signalling inhibition in the high-glucose group, α-SMA expression was significantly reduced to an undetectable level (Fig. 5g, h). These findings indicated that high glucose induces PSC activation via the TGFβ signalling pathway.

We next investigated the effect of GSH on ROS production and TGFβ signalling in PSCs. As shown in Fig. 5k, l, compared with that in the low-glucose group, ROS levels in PSCs treated with high glucose and Rosup reagent were significantly increased (*P* < 0.05). Conversely, GSH reduced excess ROS production in response to high glucose (*P* < 0.001). In addition, the increases in α-SMA and collagen I expression in response to high glucose were effectively suppressed by GSH treatment (*P* < 0.001, *P* < 0.05 respectively, Figs. 5o, p, r S2), indicating that GSH inhibited high glucose-induced PSC activation. To investigate the mechanism underlying the effect of GSH on TGFβ signalling, we next detected TGFβ1 protein expression and SMAD3 phosphorylation. The increases in TGFβ1 protein expression and SMAD3 phosphorylation induced by high glucose were significantly blocked in PSCs treated with GSH (Figs. 5m–o, q, and S2).

**Fig. 6:**
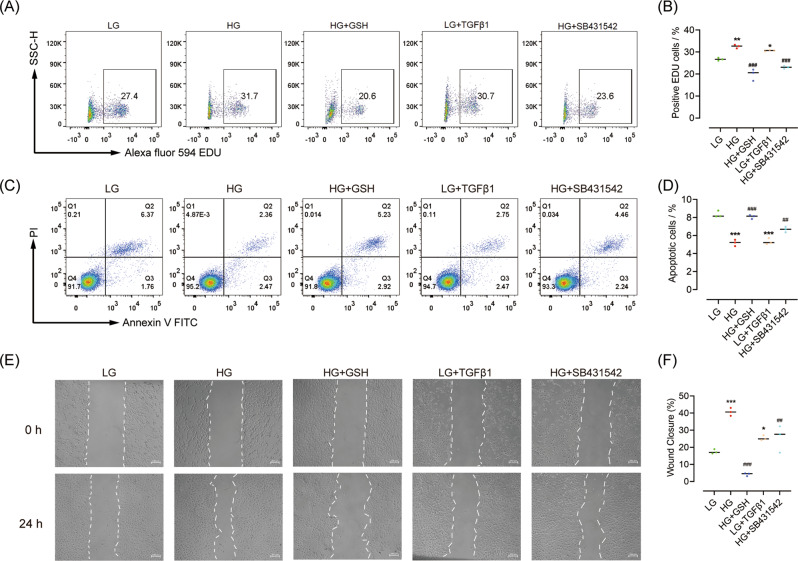
GSH inhibits high glucose-induced PSC proliferation and migration via TGFβ signalling in vitro. **A**, **B** Representative and statistical data of cell proliferation detected by flow cytometry. **C**, **D** Representative and statistical data of apoptosis detected by flow cytometry. **E**, **F** Representative images and statistical data of cell migration. *n* = 3/group; *P < 0.05, **P < 0.01, ***P < 0.001 vs. low glucose; ^##^*P* < 0.01 vs. high glucose.

Finally, we detected apoptosis, proliferation, and migration in various treatment groups. Compared with low glucose administration, high glucose and TGFβ1 enhanced the proliferation and decreased the apoptosis of PSCs, whereas GSH and SB431542 prevented these effects (Figs. 6a–d and S3). Cell migration assessment yielded similar results (Fig. 6e, f).

Taken together, the results corroborated that GSH inhibited PSC activation via down-regulation of the TGF-β/SMAD pathway.

### GSH inhibits high glucose-induced human PSC proliferation via TGFβ signalling in vitro

**Fig. 7:**
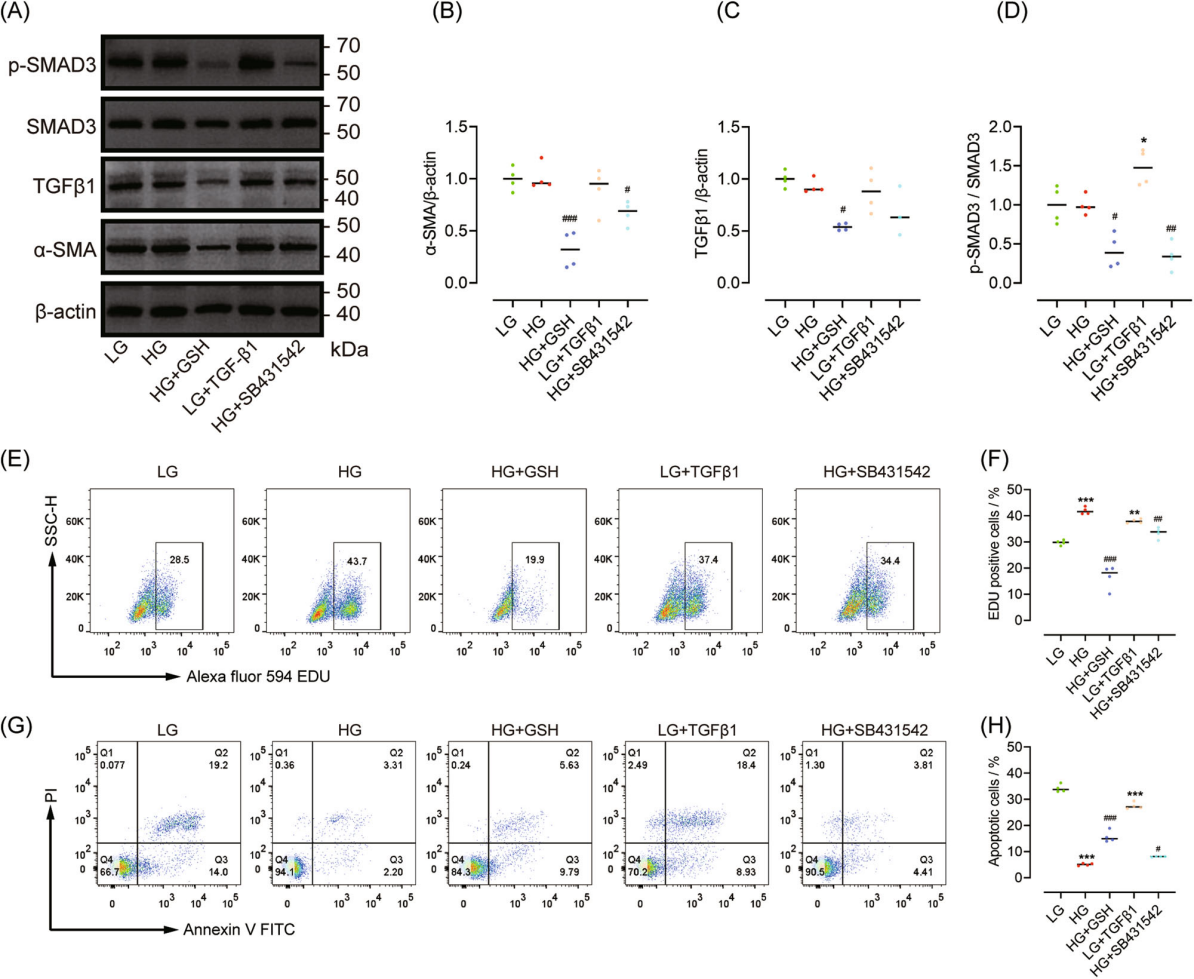
GSH inhibits high glucose-induced human PSC proliferation via TGFβ signalling in vitro. **A**–**D** Western blot data of α-SMA, SMAD3, p-SMAD3, and TGFβ1 (levels are relative to those in LG group, *n* = 4/group). **E**, **F** Representative and statistical data of cell proliferation detected by flow cytometry. **G**, **H** Representative and statistical data of apoptosis detected by flow cytometry. *n* = 4/group; *P < 0.05, **P < 0.01, ***P < 0.001 vs. low glucose; ^#^*P* < 0.05, ^##^*P* < 0.01, ^###^*P* < 0.001 vs. high glucose.

Using the activated (passaged) hPSC line, we determined the effects of GSH and SB431542 on hPSC activation and proliferation. Both GSH and SB431542 significantly down-regulated the TGFβ1/SMAD pathway and the expression of α-SMA under high glucose conditions (Fig. 7a–d). Consistent with the effects seen in rat PSCs, high glucose and TGFβ1 enhanced the proliferation and decreased the apoptosis of hPSCs under low glucose conditions, whereas GSH and SB431542 prevented these effects in the presence of high glucose (Fig. 7e–h).

## Discussion

**Fig. 8:**
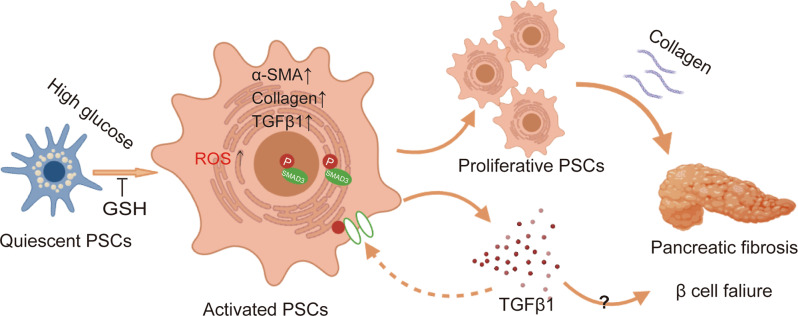
Detailed mechanism of GSH inhibiting pancreatic fibrosis induced by high glucose. GSH prevents high glucose-induced pancreatic fibrosis by suppressing PSCs activation and proliferation via the ROS/TGFβ/SMAD pathway.

The mechanism of pancreatic fibrosis in PSCs in chronic inflammation or pancreatic cancer is well known, but studies focusing on the potential role in islet fibrosis and β-cell failure are limited [10, 11]. In the present study, we established a rat model of β-cell failure using LOsG and found that high glucose promoted PSC activation and proliferation to enhance pancreatic fibrosis. GSH prevented these developments by blocking the ROS/TGFβ/SMAD signalling pathway (Fig. 8).

Insulin resistance and impaired β-cell function are hallmarks of T2DM. In the past few decades, insulin resistance has been considered the main cause of this disease [25]. However, unless β-cell failure occurs simultaneously, insulin resistance will not lead to T2DM. There are many more people with insulin resistance than people with T2DM, which indicates that insulin resistance is necessary, but not sufficient to induce the onset of diabetes [26]. Clinical studies have shown that at the time of diagnosis, β-cell mass and function are significantly reduced in patients with T2DM [27]. Therefore, β-cell failure is considered to play a major role in the pathogenesis of the disease. In the present study, we successfully established a rat model of β-cell failure through LOsG intake. Although excessive sugar intake is considered a major cause of T2DM [2, 28], the exact mechanism by which high glucose causes β-cell failure remains unclear.

There are numerous risk factors for β-cell failure. Chronic inflammation, endoplasmic reticular stress, and OxS caused by glycolipid toxicity are all related to the progression of β-cell failure [29, 30]. Pancreatic islet tissue disorder induced by fibrosis is another important mechanism of β-cell failure [7, 31]. In this study, we showed that β-cell failure induced by LOsG was accompanied with increased pancreatic fibrosis. This is consistent with previous findings that fibrosis may accelerate β-cell destruction or induce the destruction of β-cell connectivity, leading to decreased insulin secretion [23, 32]. Increasing evidence indicates that PSC activation plays an important role in the progression of islet fibrosis in T2DM patients and animal models [7, 33, 34]. In this study, we showed that LOsG significantly increased the total number of PSCs and the number of α-SMA-positive PSCs. In vitro, isolated PSCs in the high-glucose group expressed higher levels of α-SMA and collagen I and had a higher cell proliferation rate. This evidence suggested that high glucose induces the activation of PSCs and promotes their proliferation, leading to increased ECM secretion. In accordance with our results, Saito et al. have shown that inhibition of PSC activation is associated with a decrease in islet fibrosis and an increase in insulin secretion [35], indicating the potential role of PSC activation in β-cell failure.

There are numerous risk factors for PSC activation, including inflammation, OxS and, other adverse factors [9, 10]. Sustained hyperglycaemia is linked to increased OxS and an increased risk of tissue damage. Our data showed that a LOsG diet can lead to ROS accumulation in both blood cells and pancreatic tissues. ROS accumulation is positively correlated with pancreatic fibrosis in both acinar and islet tissues, confirming the role of OxS in fibrosis onset [36]. Considering that PSC activation is a key factor in pancreatic fibrosis, we speculated that excess ROS production induced by high glucose may induce PSC activation in vivo. Indeed, the numbers of total PSCs and activated PSCs were significantly increased upon LOsG treatment. This is consistent with the finding of Ryu et al. [37] that high glucose induces PSC activation by OxS. However, they did not explore the underlying mechanism. In vitro, we found that in PSCs, high ROS accumulation attributed to high glucose was associated with PSC activation, which was characterized by increased expression of α-SMA, accelerated migration, and increased synthesis of ECM proteins such as collagen I, all of which were rescued by GSH treatment.

As OxS plays an important role in PSC activation and fibrosis, the use of antioxidants to inhibit OxS is valued by researchers [36, 37]. As the major antioxidant molecule synthesized in cells, GSH plays a vital role in regulating the level of OxS. Patients with T2DM reportedly have GSH deficiency when compared to healthy controls [18]. Increasing GSH levels through precursor supplementation is a feasible intervention that directly addresses OxS in diabetes. This approach significantly suppresses tissue damage caused by OxS in T2DM patients [38]. However, the effect of GSH on PSC activation and pancreatic fibrosis remained unknown. Our study showed both in vivo and in vitro that GSH can nearly completely inhibit PSC activation and pancreatic fibrosis induced by high glucose.

Various signal transduction and gene expression systems, including TGFβ signalling, are regulated by OxS [36]. TGF-β1 is an important cytokine that induces fibrosis in organs such as the kidneys and lungs [39, 40], and can activate PSCs to induce pancreatic fibrosis [41, 42]. In this study, we found that LOsG treatment elevated the protein levels of TGFβ1, p-SMAD3, and SMAD4 in rat pancreas. Moreover, their expression was positively correlated with that of fibronectin, an ECM protein. Confirming the important role of the TGFβ/SMAD pathway in pancreatic fibrosis, we found that high glucose promoted the activation of PSCs in vitro. After the addition of TGFβ1 to the low-glucose medium, α-SMA expression in PSCs increased significantly. In contrast, after the addition of SB431542, a TGFβ signalling inhibitor, to the high-glucose medium, α-SMA expression in PSCs decreased to a nearly undetectable level. Both in vivo and in vitro, GSH treatment fully blocked TGFβ/SMAD pathway activation by inhibiting excessive ROS production. These data demonstrate that high glucose induces PSC via activation of the TGFβ/SMAD pathway.

In the activated hPSC cell line, both GSH and SB431542 significantly down-regulated the TGFβ1/SMAD pathway and the expression of α-SMA under high glucose conditions. Consistent with the effects in rat PSCs, high glucose and TGFβ1 levels enhanced the proliferation and decreased the apoptosis of PSCs in the LG group, whereas GSH and SB431542 prevented the effects induced by high glucose. These data suggest that high glucose levels promoted the proliferation of activated hPSCs through the TGFβ signalling pathway, and GSH has the potential to attenuate the activation phenotype of hPSCs.

In summary, we found that high glucose increases ROS production in PSCs, which in turn activates PSCs by upregulating the TGFβ signalling pathway to promote their proliferation and migration. Activated PSCs have an increased ability to synthesize and secrete ECM, leading to increased pancreatic fibrosis and β-cell failure. Timely intervention with the antioxidant GSH to inhibit high glucose-induced PSC activation and proliferation via blocking the TGFβ pathway is important for the prophylaxis and treatment of diabetes and may help protect people at risk of T2DM from pancreatic fibrosis and subsequent β-cell failure. Further research on human PSCs is needed to confirm the application prospects of GSH.

## Supplementary information

Supplementary table 1

Supplementary table 2

Supplementary figure 1

Supplementary figure 2

Supplementary figure 3

Supplementary figure legends

Reproducibility checklist

Original Data File

## Data Availability

The datasets used and/or analysed during the current study are available from the corresponding author on reasonable request.
