# Supplementary material for: Glutathione prevents high glucose-induced pancreatic fibrosis by suppressing pancreatic stellate cell activation via the ROS/TGFβ/SMAD pathway
Source: Cell Death Dis. 2022 May 6;13(5):440. doi: 10.1038/s41419-022-04894-7 (PMC9076672; doi:10.1038/s41419-022-04894-7)
Supplement: Supplementary file 1 — Supplementary table 1 [file 41419_2022_4894_MOESM1_ESM.docx]

**Supplementary table 1.** Sequences of the primers used for RT-qPCR.

| **Gene** | **Forward primer (5'-3')** | **Reverse primer (5'-3')** |
| --- | --- | --- |
| Insulin | CCTGCCCAGGCTT TTGTCA | GGTGCAGCACTGATCCACAATG |
| α-SMA | ATGCCTCTGGACGTACAACTG | CACACCATCTCCAGAGTCCA |
| TGFβ1 | GACTCTCCACCTGCAAGACC | GGACTGGCGAGCCTTAGTTT |
| Collagen I | ACTGGCAACCTCAAGAAGTCCC | AAGTTCCGGTGTGACTCGTGC |
