# Supplementary material for: Glutathione prevents high glucose-induced pancreatic fibrosis by suppressing pancreatic stellate cell activation via the ROS/TGFβ/SMAD pathway
Source: Cell Death Dis. 2022 May 6;13(5):440. doi: 10.1038/s41419-022-04894-7 (PMC9076672; doi:10.1038/s41419-022-04894-7)
Supplement: Supplementary file 2 — Supplementary table 2 [file 41419_2022_4894_MOESM2_ESM.docx]

**Supplementary table 2.** Primary antibodies used for immunostaining and western blotting.

| **Name** | **Species** | **Vendor information** | **Catalog number** | **Application and dilution** |
| --- | --- | --- | --- | --- |
| Insulin | Mouse | Proteintech  (Wuhan, China) | 66198-1-Ig | 1:1000 (IF) |
| Insulin | Rabbit | Proteintech | 15848-1-AP | 1:500 (IF) |
| Glucagon | Rabbit | Boster (Wuhan, China) | BM1621 | 1:200 (IF) |
| Fibronectin | Mouse | Proteintech | 66042-1-Ig | 1:200 (IF), 1:5000 (WB) |
| Collagen I | Rabbit | Proteintech | 14695-1-AP | 1:5000 (WB) |
| Desmin | Rabbit | Proteintech | 16520-1-AP | 1:200 (IF) |
| α-SMA | Rabbit | Proteintech | 14395-1-AP | 1:2000 (WB) |
| α-SMA | Mouse | Proteintech | 67735-1-Ig | 1:400 (IF) |
| GFAP | Mouse | Cell Signaling Technology (MA, USA) | 3670T | 1:100 (IF) 1:1000 (WB) |
| Vimentin | Rabbit | Proteintech | 10366-1-AP | 1:50 (IF) |
| TGFβ1 | Rabbit | Proteintech | 21898-1-AP | 1:50 (IF), 1:2000 (WB) |
| SMAD3 | Rabbit | Huabio  (Hangzhou, China) | ET1607-41 | 1:1000 (WB) |
| p-SMAD3 (S423/S425) | Rabbit | Huabio | ET1609-41 | 1:500 (WB) |
| SMAD4 | Rabbit | Beyotime  (Shanghai, China) | AF1291 | 1:1000 (WB) |
| β-actin | Mouse | Proteintech | 66009-1-Ig | 1:10000 (WB) |
| α-tubulin | Mouse | Proteintech | 66031-1-Ig | 1:5000 (WB) |

IF: immunofluorescence; WB: western blotting.
