# Supplementary figures and images for: Glutathione prevents high glucose-induced pancreatic fibrosis by suppressing pancreatic stellate cell activation via the ROS/TGFβ/SMAD pathway
Source: Cell Death Dis. 2022 May 6;13(5):440. doi: 10.1038/s41419-022-04894-7 (PMC9076672; doi:10.1038/s41419-022-04894-7)

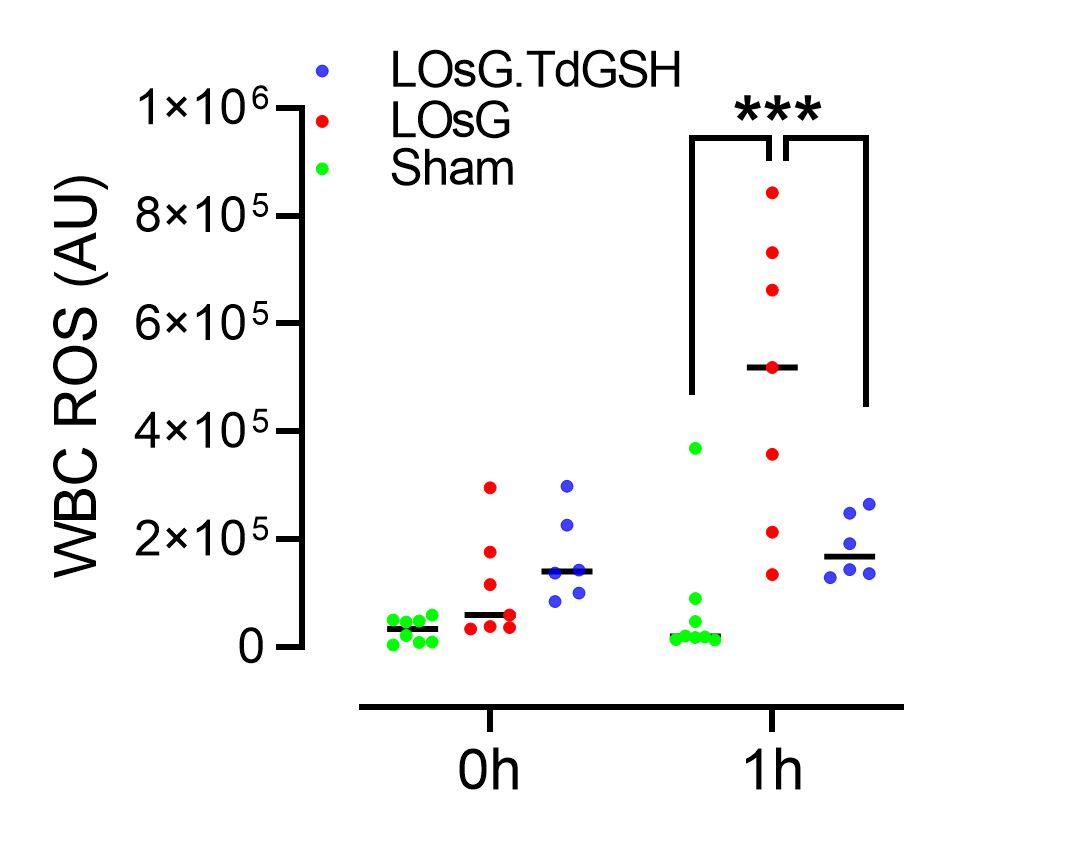

Supplement: Supplementary file 3 — Supplementary figure 1 [file 41419_2022_4894_MOESM3_ESM.tif]

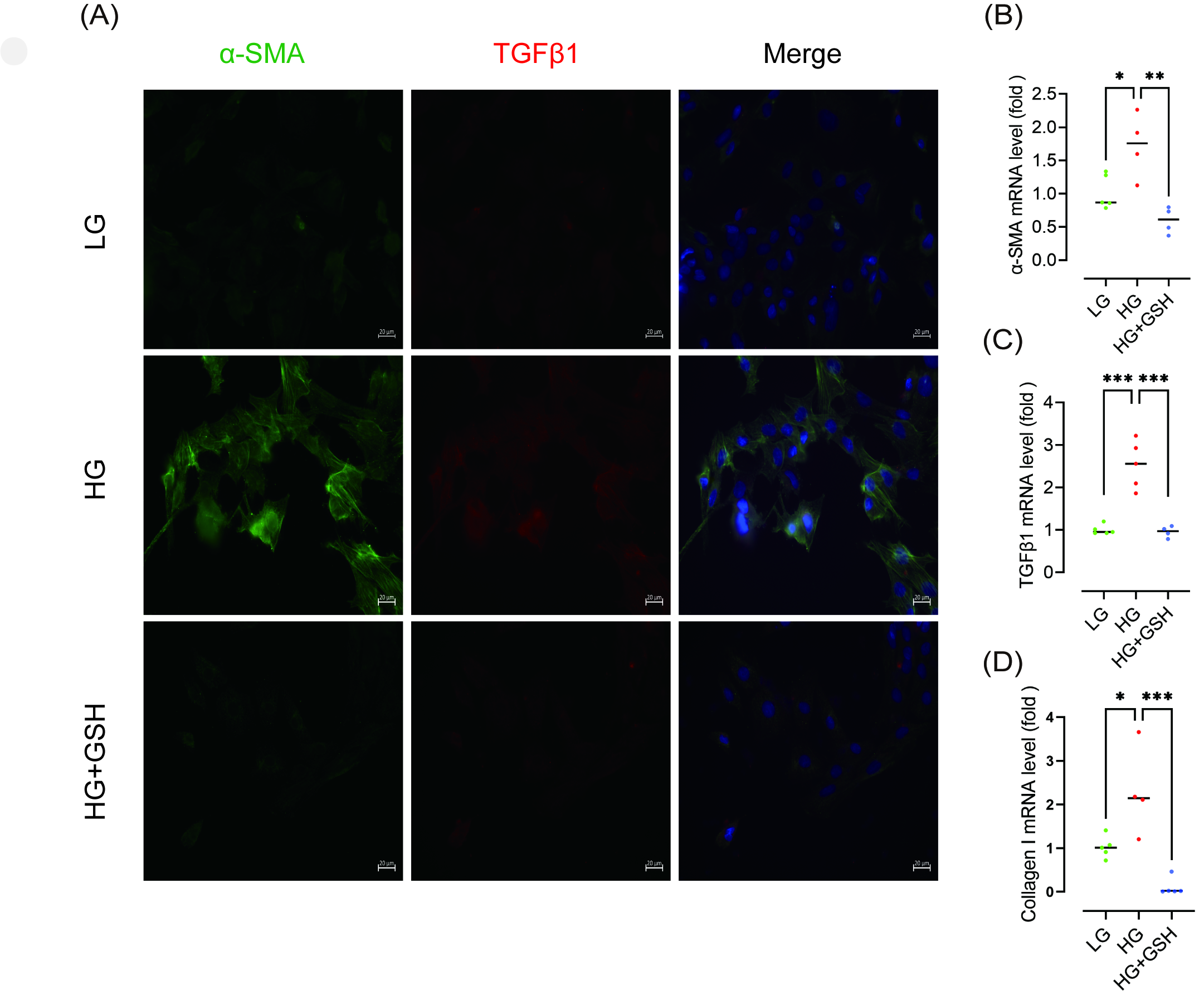

Supplement: Supplementary file 4 — Supplementary figure 2 [file 41419_2022_4894_MOESM4_ESM.tif]

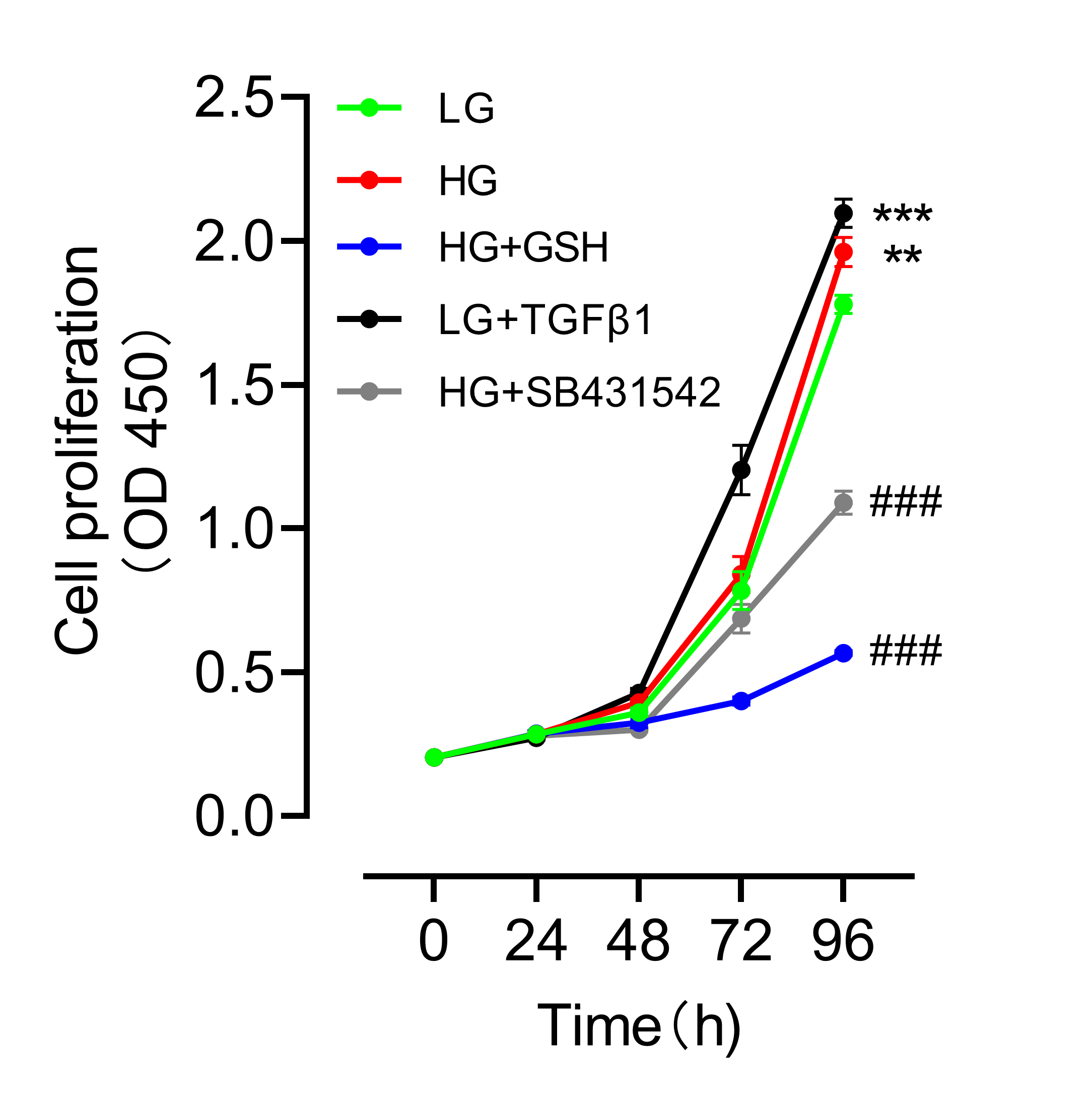

Supplement: Supplementary file 5 — Supplementary figure 3 [file 41419_2022_4894_MOESM5_ESM.tif]
