# Supplementary material for: Glutathione prevents high glucose-induced pancreatic fibrosis by suppressing pancreatic stellate cell activation via the ROS/TGFβ/SMAD pathway
Source: Cell Death Dis. 2022 May 6;13(5):440. doi: 10.1038/s41419-022-04894-7 (PMC9076672; doi:10.1038/s41419-022-04894-7)
Supplement: Supplementary file 6 — Supplementary figure legends [file 41419_2022_4894_MOESM6_ESM.docx]

**Supplementary figure legends**

**Supplementary Figure 1. GSH inhibits ROS production induced by LOsG in white blood cells (WBCs).** Quantification of ROS in WBCs (0 h and 1 h after glucose gavage). n = 6–8/group, ****P* < 0.001.

**Supplementary Figure 2. GSH inhibits high glucose-induced** **α-SMA, collagen I, and TGFβ1 expression in PSCs.** (A) Representative images of immunofluorescence staining of TGFβ1 (red) and α-SMA (green). Nuclei were stained with DAPI (blue). (B-D) mRNA levels of a-SMA, collagen I, and TGFβ1 in PSCs. Levels are expressed relative to that in sham rats. n = 4–5/group, **P* < 0.05, ***P* < 0.01, ****P* < 0.001.

**Supplementary Figure 3. GSH inhibits PSC proliferation via TGFβ signaling.** Proliferation of cultured PSCs as analyzed by the CCK‐8 assay. Data are shown as mean ± SEM (n = 8/group), ***P* < 0.01, ****P* < 0.001 vs. low glucose; ^###^*P* < 0.001 vs. high gluose.
