# Supplementary material for: Glutathione prevents high glucose-induced pancreatic fibrosis by suppressing pancreatic stellate cell activation via the ROS/TGFβ/SMAD pathway
Source: Cell Death Dis. 2022 May 6;13(5):440. doi: 10.1038/s41419-022-04894-7 (PMC9076672; doi:10.1038/s41419-022-04894-7)
Supplement: Supplementary file 8 — Original Data File [file 41419_2022_4894_MOESM8_ESM.pdf]

Glutathione prevents high glucose-induced pancreatic fibrosis by suppressing pancreatic stellate cell activation via the ROS/TGFβ/SMAD pathway

Jitai Zhang, Juan Bai, Qian Zhou, Yuxin Hu, Qian Wang, Lanting Yang, Huamin Chen, Hui An, Chuanzan Zhou, Yongyu Wang, Xiufang Chen and Ming Li

Supplementary Original Western Blots

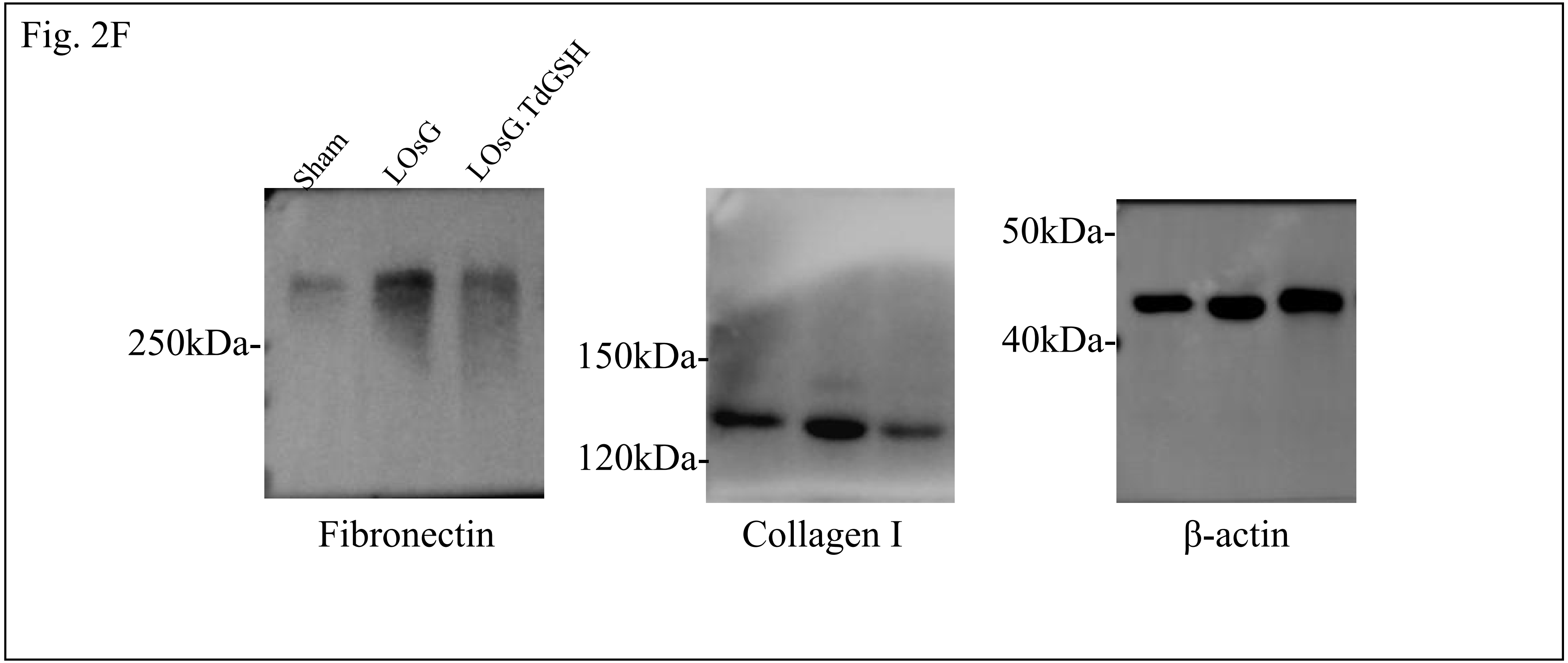

Fig. SO1 Original western blots for the western blot result in Fig. 2

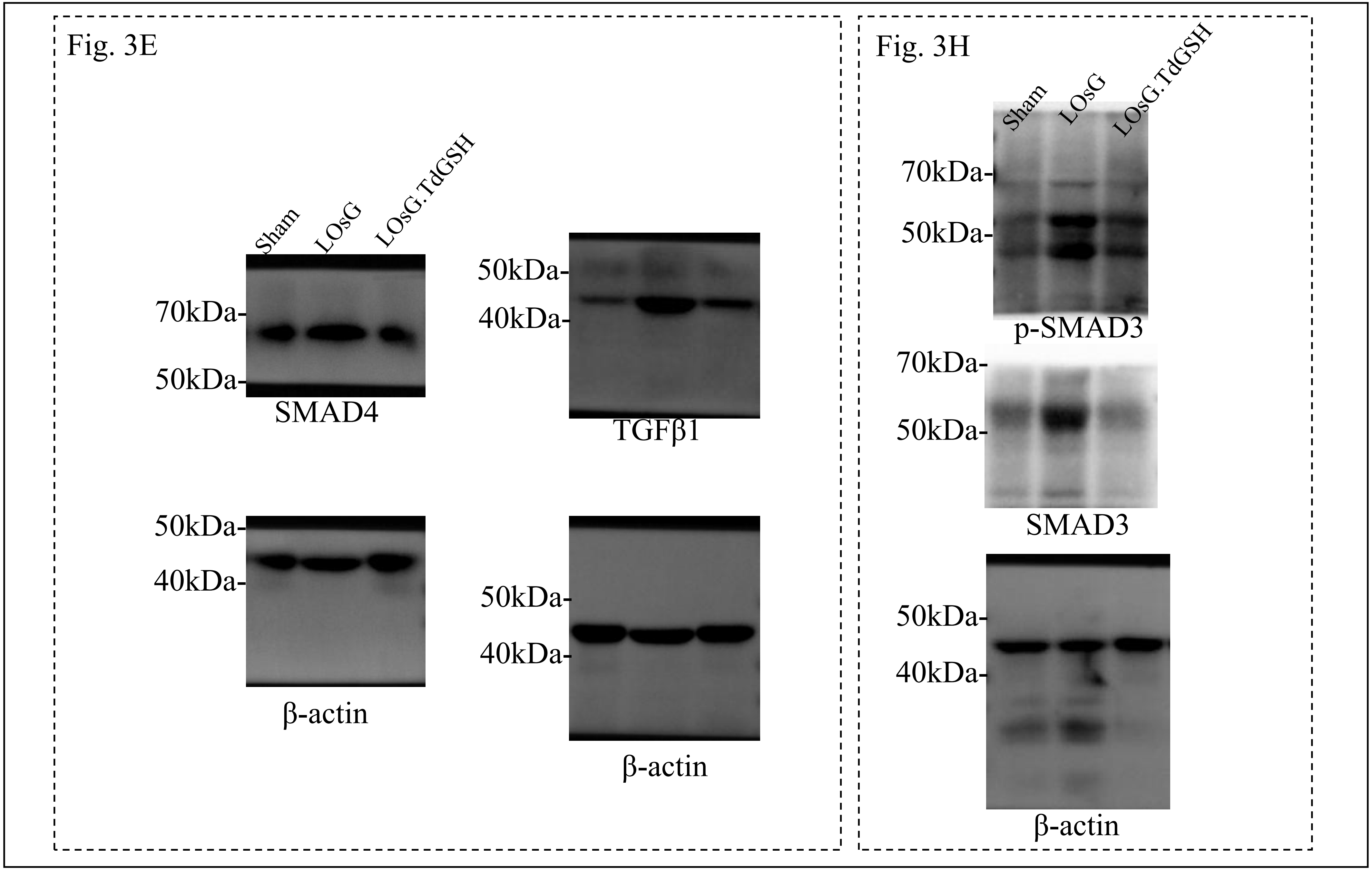

Fig. SO2 Original western blots for the western blot result in Fig. 3

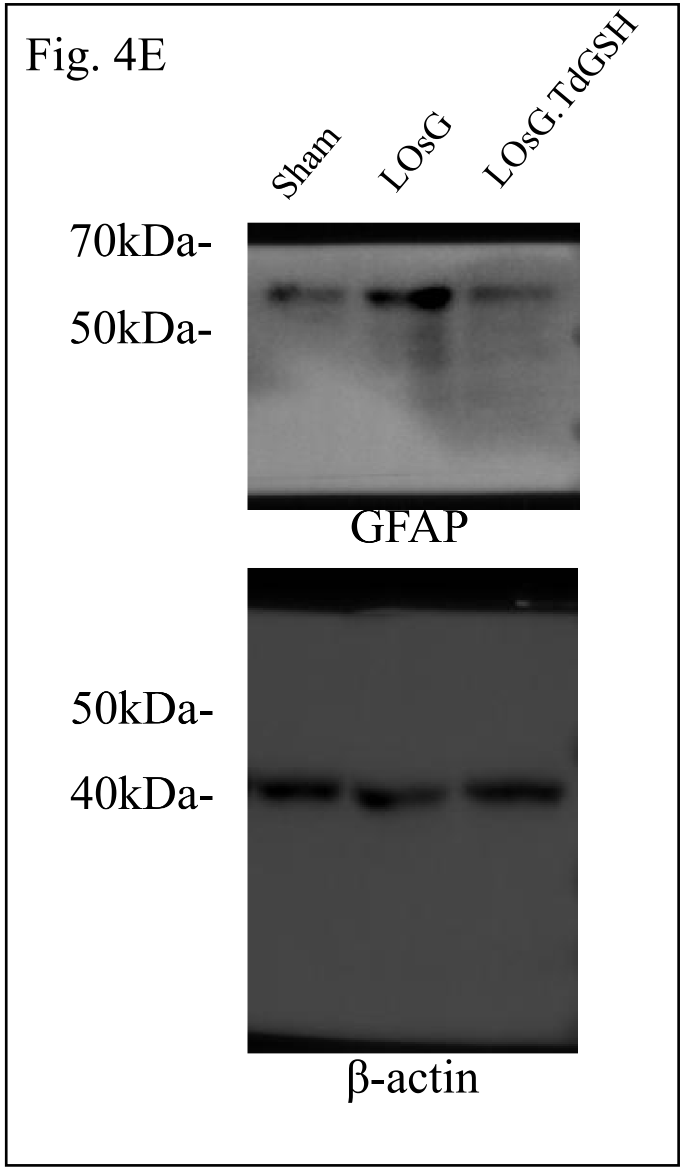

Fig. SO3 Original western blots for the western blot result in Fig.4

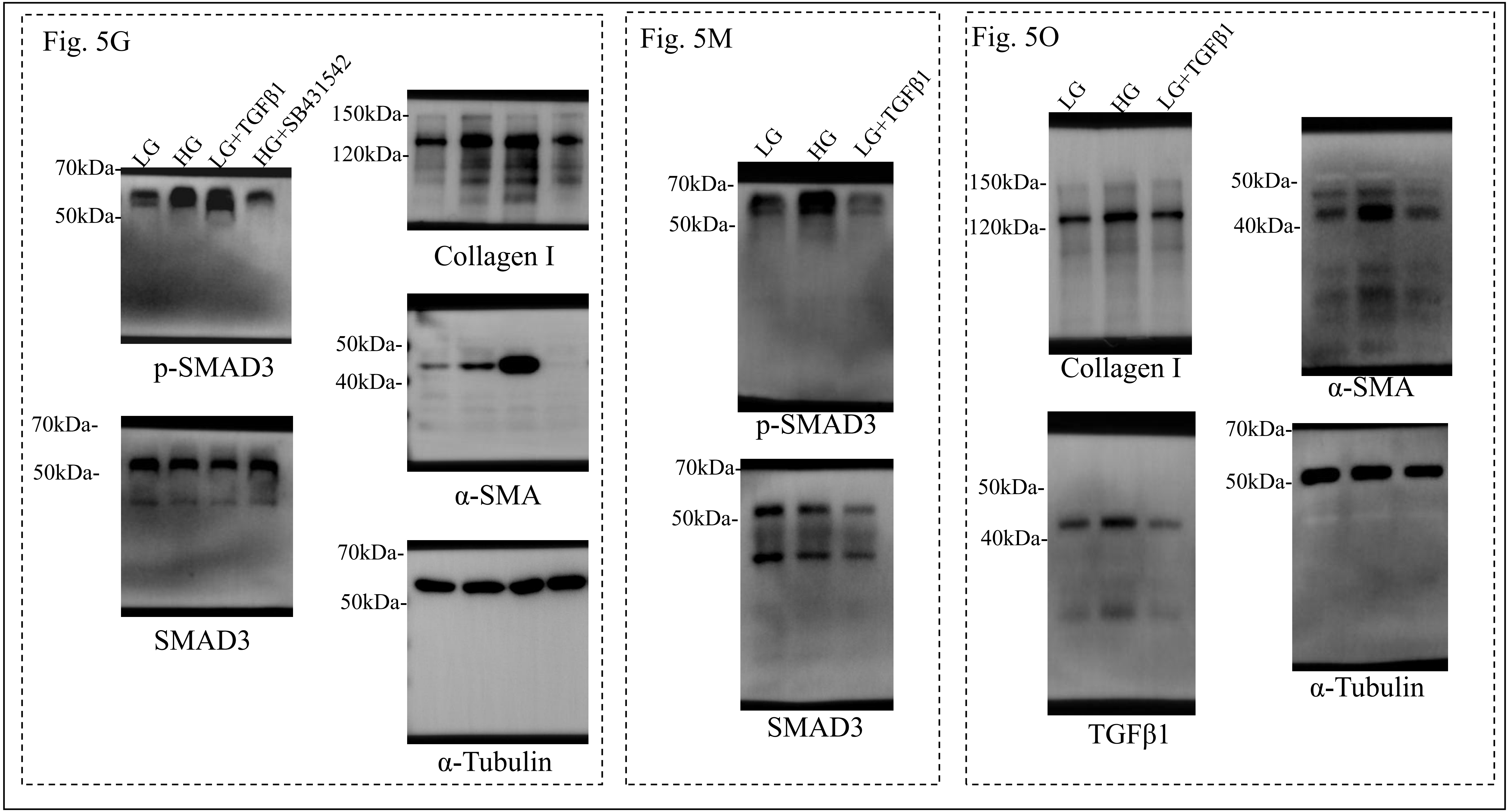

Fig. SO4 Original western blots for the western blot result in Fig. 5

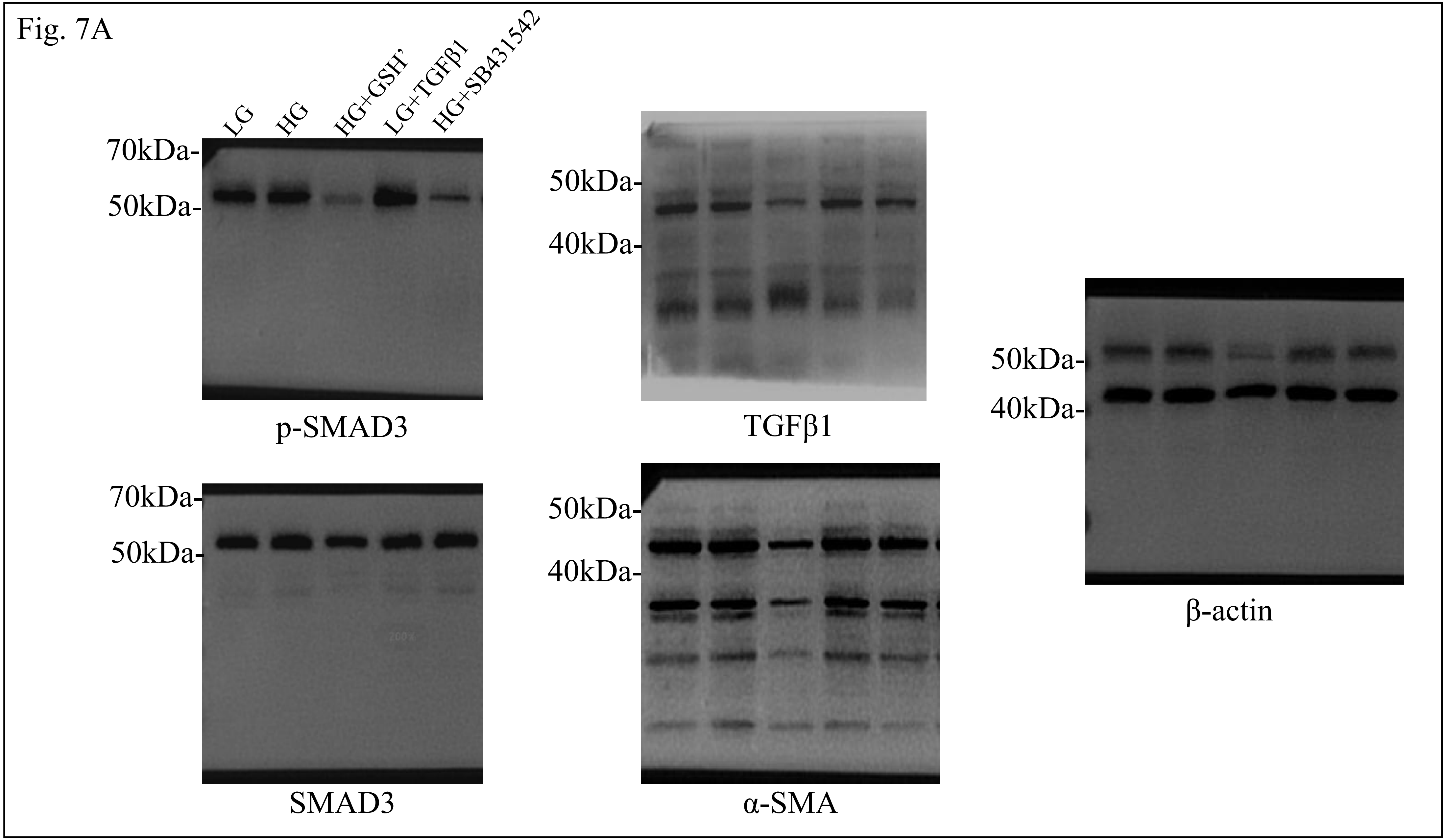

Fig. SO5 Original western blots for the western blot result in Fig. 7
